# Metabolomics as a Tool to Understand Nano-Plant Interactions: The Case Study of Metal-Based Nanoparticles

**DOI:** 10.3390/plants12030491

**Published:** 2023-01-21

**Authors:** Sónia Silva, Maria Celeste Dias, Diana C. G. A. Pinto, Artur M. S. Silva

**Affiliations:** 1 LAQV-REQUIMTE, Department of Chemistry, University of Aveiro, 3810-193 Aveiro, Portugal; 2 Centre for Functional Ecology, Department of Life Sciences, University of Coimbra, Calçada Martim de Freitas, 3000-456 Coimbra, Portugal

**Keywords:** impact on plants, metabolic pathways, metabolite content, metallic nanoparticles

## Abstract

Metabolomics is a powerful tool in diverse research areas, enabling an understanding of the response of organisms, such as plants, to external factors, their resistance and tolerance mechanisms against stressors, the biochemical changes and signals during plant development, and the role of specialized metabolites. Despite its advantages, metabolomics is still underused in areas such as nano-plant interactions. Nanoparticles (NPs) are all around us and have a great potential to improve and revolutionize the agri-food sector and modernize agriculture. They can drive precision and sustainability in agriculture as they can act as fertilizers, improve plant performance, protect or defend, mitigate environmental stresses, and/or remediate soil contaminants. Given their high applicability, an in-depth understanding of NPs’ impact on plants and their mechanistic action is crucial. Being aware that, in nano-plant interaction work, metabolomics is much less addressed than physiology, and that it is lacking a comprehensive review focusing on metabolomics, this review gathers the information available concerning the metabolomic tools used in studies focused on NP-plant interactions, highlighting the impact of metal-based NPs on plant metabolome, metabolite reconfiguration, and the reprogramming of metabolic pathways.

## 1. Introduction

Currently, nanotechnology has a key role in many fields and is a multi-million-dollar industry, with a global market size valued at 1.76 billion dollars in 2020 and estimated to reach $33.63 billion by 2030 [1]. Nanoparticles (NPs), the forefront of nanotechnology, can be found in many daily products such as cosmetics, food, textiles, and pharmaceutics, with additional uses in medicine, energy, electronics, construction, environmental remediation of contaminated soil and water, and agriculture [2,3,4,5,6,7]. This wide use of NPs makes it impossible to avoid the interaction of living organisms with such materials, which is crucial for understanding the impact that NPs may have on plants, animals, and humans.

The interaction of metal-based NPs with plants has been analyzed, at the first stage, using a toxicological point of view [8,9,10,11,12]. However, lately it has changed towards a perspective of plant fortification, stress mitigation, and plant response modulation [4,13,14,15,16,17]. On the other hand, modern agriculture is changing into precision agriculture to maximize the gains from available resources. Given their characteristics, NPs can play an important role in achieving the precision and sustainability that modern agriculture requires as they can reduce nutrient losses, reduce the amounts of agrochemicals used in plant protection and nutrition, reduce environmental risks, and minimize production costs [17]. Nevertheless, one should not put aside the potential negative impacts of NPs on agricultural systems as NPs are prone to induce phytotoxicity under certain exposure conditions. Thus, several factors, such as NP physiochemical properties, concentration, treatment duration, exposure route, and crop species must be considered for efficient, safe and sustainable use of agro-nanotechnologies.

The impact of NPs on plants has been extensively evaluated using agronomical and physiological traits and less using metabolomic and molecular tools, despite the increasing number of studies on metabolomics in recent years, thus limiting the mechanistic understanding of NPs’ mode of action and plants’ response to them [18]. Metabolomics reflects the plant status at a given point, enables the detection of changes imperceptible by phenotype-based biomarkers, and, as metabolites are the final product of gene and protein expression despite the metabolome not being fully associated with the plant genome, enlightens about NP impact on gene/protein regulation [19,20,21]. Metabolomics can be used for targeted (monitor specific metabolites) or untargeted (global metabolite detection) analysis and the most common analytical techniques used in assessing NP impact on plants are Nuclear Magnetic Resonance (NMR) spectroscopy and Mass Spectrometry (MS) coupled with chromatographic separation [20].

The potential application of metallic NPs in agriculture [4,22,23] has been recently reviewed, as well as their positive/negative impacts on plants in terms of plant physiology and biochemistry [12,15,24,25,26,27]; nevertheless, it is missing a review centered on NP impact on plant metabolomics. Thus, here we review the works available focused on plant metabolic rearrangement induced by metallic NPs, highlighting the altered metabolites and the modified metabolic pathways to unveil the NPs’ mode of action. This approach will allow readers to gain comprehensive knowledge about the metabolic pathways targeted by the metal-based NPs in diverse plant species, and key knowledge to understanding the real impact of metallic NPs on plants.

## 2. Metabolomics Applied to Understand the Biochemistry of Nano-Plant Interaction

The successful application of nanoparticles in quite a few areas contributes to their environmental release [28] and, consequently, to their interaction with plants. Moreover, conventional agriculture will soon be substituted by more sustainable agriculture. In that regard, the use of NPs is a good approach and is emerging [29,30,31]. These factors prompted some authors to investigate the effects of NPs on plants [32,33,34,35,36] as well as any adverse effects that can arise for human health [32,34,37]. Consequently, several researchers focused their research on methodologies to measure nanoparticles in the environment [38], particularly in soil and plants [39,40], and evaluate their impact on plant morphology and physiology [41,42,43]. Although these topics are of extreme importance, our primary focus is the NPs’ effect on specialized metabolite production [44] and, consequently, their impact on the plant’s response to the stressor, NP elicitation, and nutritional or medicinal value. As it is known, these metabolites are produced by plants to ensure their adaptation and/or survival to the surrounding conditions [45], but they are also associated with health-promoting bioactivities [46]. Therefore, metabolomics [21,47,48,49], a technique that identifies and quantifies all low-weight metabolites, is an excellent technique to study the nanoparticles’ impact on plant-specialized metabolite production. At the moment, the most common analytical tools for metabolomics involve NMR spectroscopy and MS, the last one coupled with chromatographic techniques such as gas and liquid chromatography [50,51].

### 2.1. Analytical Methods

NMR is a nondestructive technique that can be used in qualitative and quantitative analysis and has high reproducibility [52,53,54]. The main advantages of NMR include the easy handling of the samples, the release of crucial structural information that allows the identification of the metabolites, and the performance of in vivo studies through isotope tracing [53,54,55]; it is also not limited to liquid samples [56]. Different NMR experiments with several levels of correlation can be performed simultaneously with the analysis of different nuclei, including some that are biologically relevant such as ^1^H, ^13^C, and ^15^N. Some important experiments used in metabolomic analyses include 1D and 2D NMR, correlated spectroscopy, total correlation spectroscopy, and heteronuclear single-quantum spectroscopy. The main disadvantage is its low sensitivity, thus requiring high concentrations [57]. However, 2D techniques can improve the NMR sensitivity and reduce the acquisition times [54,55]. Although several nuclei can be analyzed and used to establish the sample profile, the most common one is ^1^H [52], primarily due to its abundance and easy use in quantifications; furthermore, the NMR coupling with LC allows the separation of the sample compounds simultaneously with ^1^H spectrum acquisition, which improves the phytochemical analysis [55].

Although MS is destructive [58], it is the most common analytical technique for metabolomic analysis, due to its high sensitivity and suitability for high-throughput analysis [59]. MS-based methods are based on the monitoring of mass-to-charge ratios (*m/z*) of all ionizable molecules present in a sample, and quantification is usually performed using standard calibration curves [60]. In addition, different separation techniques can be coupled to MS, such as LC, GC, and capillary electrophoresis. However, LC-MS and GC-MS are the most common due to their versatility and robustness. The main advantages of GC-MS over LC-MS are the high chromatographic resolution and reproducible retention times [51].

GC is usually coupled to hard ionization sources such as electron impact ionization, allowing in-source fragmentation and identification of the molecular ion with the extensive databases available for GC-MS [61]. However, the use of GC-MS is limited to volatile analytes; to detect nonvolatile compounds such as amino acids, sugars, and organic acids; derivatization is required before GC-MS can analyze them [62]. Alternatively, LC-MS provides high sensitivity and selectivity for analyzing nonvolatile compounds. However, separation by LC is susceptible to retention time shifts [63]. The use of 2D LC and GC has also been implemented for metabolomics to minimize interferences from complex matrices to increase chromatographic resolution and peak capacity [64].

### 2.2. Metabolite Extraction and Analytical Methods Used in Metallic NP Studies

Several metabolomic techniques have been used to unravel the metabolite changes induced by metallic NPs in plants. Nevertheless, the main analytical methods rely on LC-MS and GC-MS, which can be or not be coupled with a time-of-flight mass spectrometer (TOF-MS). Most of the works use fresh [18,65,66,67,68,69], dry [70,71,72], freeze-dried [73,74,75,76,77], or frozen sample material [19,78,79,80] that is then homogenized with the solvent for metabolite extraction. For LC-MS analysis, most of the studies used methanol: formic acid [73,76] or methanol: water [13,65], or only methanol [79] or water [81]. Sun et al. [82] used perchloric acid: NaOH: benzoyl chloride: NaCl: anhydrous ether: methanol or chloroform: methanol as solvents for targeted metabolomics. For GC-MS, the metabolite extraction is usually performed using a mixture of methanol: chloroform: water [19,77,78,80,83], methanol: water [68,71,84], chloroform: hexane: methanol [85], isopropanol: acetonitrile: water [74,75], or only methanol [67,69], or hexane [66]. For the analysis of root exudates, metabolites were extracted with water: ethyl acetate: chloroform [86]. After metabolite extraction for GC-MS, samples are usually derivatized using a two-step procedure: oximation followed by silylation [74,78,84,86].

## 3. Understanding the Impact of Metal-Based Nanomaterials on Plant Metabolite Reconfiguration

NPs cover a wide range of materials, but few have been extensively studied. For instance, the ones most investigated for their impact on plants are the NPs most commonly used in industries (such as metal and metal oxide NPs of titanium dioxide, silver, zinc oxide, cerium dioxide, copper, copper oxide, aluminum, nickel, and iron) [4,27]. These NPs can act as abiotic stressors inducing stimulatory effects or phytotoxic symptoms, depending on their origin/material and concentration [79,87]. They can affect plants at several levels, inducing metabolite reconfiguration, physiological and morphological alterations, and genotoxic changes [4,27,78,88].

Within the metabolite adjustments, metabolomic studies on the effects of NPs have yet to be addressed; moreover, the few studies available indicate changes in primary and secondary metabolism [78,79,89]. These changes are mostly related to the overproduction of reactive oxygen species (ROS) induced by the nanomaterials [90], which can induce oxidative stress, depending on several factors such as plant stress tolerance, age, vegetative stage, and type of tissues [26].

### 3.1. The Tenuous Line between the Potential Toxicity and the Benefits of Metal-Based NPs

Metal-based NPs have been reported to cause phytotoxic effects on plants, decreasing their growth and performance [88,91]. One of the most commonly described symptoms of NP phytotoxicity is the accumulation of ROS and secondary signaling messengers, leading to transcriptional regulation of several metabolic pathways [29]. In turn, the overproduction of ROS can result in oxidative stress and, directly or indirectly, affect primary and secondary metabolite production [89]. Yet, the toxicity of the NPs depends on several factors, such as the nature of the material, their size, the concentration used, and the exposure scenario [90].

Despite the adverse effects caused by the exposure of plants to NPs, several works have highlighted the positive impact on plant growth and stress tolerance [4,92]. For instance, treating several species, including crops and ornamentals, with metal-NPs is reported to modulate the profile of primary and secondary metabolites [89]. This metabolite reconfiguration can promote photosynthetic reactions and growth, improve the plant antioxidant defense response [4,87], and increase plant tolerance to abiotic stresses [68,93,94].

Considering the contrasting effects on plants is thus critical to accurately assess the potential risks vs. benefits of engineered nanomaterial application in agrosystems. With that said, omics is an indispensable tool to fully understand the mode of action of the NPs and the molecular and metabolic targets that lead to the contrasting morphological and physiological effects described in the literature.

#### 3.1.1. Titanium Dioxide Nanoparticles (nTiO_2_)

Titanium dioxide nanoparticles (nTiO_2_) are widely used and explored by distinct industries such as pharmaceutics, medicines, coating, inks, plastics, food, cosmetics, textiles, solar cells, agriculture, environmental remediation, and more applications are being pursued [95]. A range of morphological, physiological, and biochemical effects on plants, sometimes conflicting, were already described: growth impairment [77,96] vs. improvement [97,98,99], an increase in chlorophyll content [11,97] vs. decrease [11,100]; photosynthesis decay [91] vs. stimulation [98]; ROS accumulation and/or antioxidant response disturbance [11,77,88] vs. redox status maintenance [99]; no physiological alterations [101] vs. geno/cytotoxicity [10,11,102]. Titanium dioxide nanoparticles (nTiO_2_) are one of the most studied metallic NPs in terms of its phytotoxicity and potential to alleviate plant stress. Thus, it is not surprising that metabolomic studies are also some of the most addressed, as summarized in Table 1.

Metabolomic studies conducted with wheat plants grown in hydroponics and exposed to nTiO_2_ (5, 50, and 150 mg L^−1^) confirmed that metabolic and physiological changes were associated with oxidative stress and antioxidant defense system activation [78,88]. These NPs acted on the phenylalanine and tryptophan pathways, amino acids, and glycerolipid biosynthesis in the glutathione-ascorbate cycle and also up-regulated tocopherol production. High doses of TiO_2_-NPs affected the biosynthesis of sugars and, consequently, the tricarboxylic acid (TCA) cycle to a greater extent. In rice (*Oryza sativa* L.) plants grown hydroponically, higher nTiO_2_-NPs doses (100, 250 and 500 mg L^−1^) shifted the metabolism towards the energy metabolism and the synthesis of antioxidants to cope with nTiO_2_ toxicity [77]. This metabolite adjustment resulted in an increase in glucose-6-phosphate, glucose-1-phosphate, succinic acid, and isocitric acid, but in inhibition of sucrose, isomaltulose, and glyoxylic acid production [77]. Nevertheless, most of the amino acids, fatty acids, and secondary metabolites that correlated with crop quality increased [77].

In *Cucumis sativus* plants, a foliar spray with 2.7 or 27 mg plant^−1^ nTiO_2_ reported an improvement of the photosynthetic rate, together with the decrease in leaf lipid peroxidation, the increase in total phenolic content, and alterations in the leaf metabolite profile of amino acids, organic acids, fatty acids, sugars, and alcohols [80]. The lowest dose had a minimal impact on cucumber metabolism; nevertheless, a total of 31 metabolites were up- or down-regulated in the highest dose, demonstrating a dose-related response [80]. The relative abundance of amino acids such as 3-hydroxy-L-proline, norvaline, *N*-ethylglycine, glutamine, 5-aminovaleric acid, and phenylacetaldehyde was increased, whereas *N*-acetyl-5-hydroxytryptamine and 2-amino-2-norbornanecarboxylic acid were reduced, suggesting adjustments in protein biosynthesis and nitrogen metabolism [80]. Authors also pinpointed alterations in carbohydrate metabolism, nutrient transport and transformation, and photosynthesis as a consequence of organic and fatty acid reconfiguration, where 2-ketobutyric acid, 3-hydroxyflavone, 2-furoic acid, phytanic acid, and pelargonic acid were enhanced, and succinic acid, tartaric acid, and glucoheptonic acid reduced [80]. These results clearly show how nTiO_2_ can disturb plant metabolism and induce stress responses involving several metabolic pathways.

Soil amended with 25–750 mg kg^−1^ nTiO_2_ promoted rice plant growth and significantly altered the metabolite profile in grains of plants grown under 750 mg kg^−1^ nTiO_2_ [68]. Several compounds augmented (proline, aspartic acid, glutamic acid, palmitic acid, glycerol, inositol, ribitol, phosphoric acid, and glycerol-3-phosphate proline, aspartic acid and glutamic acid), whereas overall fatty acids (linoleic and oleic acid), organic acids, and sugar contents decreased [68]. Changes in carbohydrate metabolism were also described in *Zea mays* plants grown in soil amended with 100 mg kg^−1^ nTiO_2_, as well as alterations in the inositol phosphate metabolism, ascorbate/aldarate, methane, glyoxylate and dicarboxylate, and TCA cycle, apart from alterations in nitrogen metabolism with changes in amino acid pools and nitrogen-containing compounds (e.g., 4-aminobutyric acid and its precursor glutamic acid, and putrescine) [19].

#### 3.1.2. Cerium (IV) Oxide Nanoparticles (nCeO_2_)

Cerium (IV) oxide nanoparticles (nCeO_2_) are highly manufactured NPs with extensive commercial applications, have residual dissolution in the environment, and thus are predicted to persist in soil, interacting with plants in their nano-sized form [106]. It is known that nCeO_2_ can alter plant growth [107,108], plant physiology, namely the antioxidant response [109,110,111], photosynthesis [43,112], and nutritional composition [113]. Furthermore, they can be assimilated and accumulated in plants [114,115]. Besides physiology, nCeO_2_ can also readjust the metabolome in plants, as listed in Table 2.

In rice seedlings germinated in 5 mL nCeO_2_ suspensions, Rico et al. [66] reported an increment in lauric and valeric acid content in plants treated with 150 mg L^−1^ nCeO_2_ together with an increment in lipid peroxidation. In the same seedlings, but now treated with 500 mg L^−1^, nCeO_2_ decreased lignin content and increased H_2_O_2_ content despite the marked increment in peroxidases and glutathione reductase activity. On the other hand, in seedlings treated with 62.5 mg L^−1^ nCeO_2_, lauric acids increased and membrane damage was maintained under control. These results highlight the potential of nCeO_2_ in inducing oxidative stress for doses above 150 mg L^−1^ [66,117]. Another study analyzed the effect of nCeO_2_ on the compositional fingerprint of root xylem in wheat, rice, and barley seedlings germinated in 5 mL nCeO_2_ suspensions [118]. It was found that the exposure to 125–500 mg L^−1^ nCeO_2_ in rice and wheat, and to 62.5–250 mg L^−1^ in barley, induced significant spectral variability in xylem with compounds such as protein, cellulose, and lipids being the most affected [118].

*Phaseolus vulgaris* plants, grown in agar medium supplemented with low doses of nCeO_2_ (25, 50, and 100 mg L^−1^), were positively impacted by all NP doses, improving plant fresh and dry weight, were responsive to 25 mg L^−1^ nCeO_2_ regarding proline (increased in shoots but not in roots), and showed a metabolic response dependent on the dose in both roots and leaves [79]. This study reported the stimulation of the secondary metabolism: terpenes and terpenoids were responsive to the treatments (steroids, steroid hormones, di and triterpenes, and carotenoids best represented the isoprenoid family); alkaloids such as 1-*O*-caffeoyl-*ß*-*D*-glucose, esculin, and (+)-sesamolinol were particularly affected; flavonoid profile was severely altered and was dose-dependent; compounds involved in the glucosinolate pathway and phytoalexin biosynthesis were up-accumulated [79]. These results show that the response induced by nCeO_2_ exposure can be related to oxidative stress, a hypothesis supported by the increase in glutathione andδ-tocotrienol, both involved in the antioxidant response, and of jasmonates, known to be involved in plant defense responses and secondary metabolite increase [79]. Nevertheless, hormones such as gibberellins, auxins, and brassinosteroids were also up-accumulated, which can justify the changes in phenotype as they are involved in plant physiological responses, and support the presence of stress induced by nCeO_2_, since brassinosteroids in particular are involved in the regulation of signal transduction pathways to stimulate stress tolerance [79,119].

Soil amendment with nCeO_2_ altered the protein content in *C. sativus* fruits exposed to 400 mg kg^−1^ nCeO_2_: globulin increased, while glutelin decreased [116]. On the other hand, when exposed to 800 mg kg^−1^ nCeO_2_, a decrease in phenolic content together with the increase in total sugar content was detected, impacting the nutritional quality of the fruit [116]. In *T. aestivum* plants, soil amendment with 125–500 mg kg^−1^ nCeO_2_ promoted plant growth and improved yield parameters under the highest dose, whereas the lowest dose changed the amino acid and fatty acid profiles in grains: 125 mg kg^−1^ nCeO_2_ increased the content of arginine, aspartic acid, glycine, histidine, and lysine, showing a trend in improvement of amino acid content in wheat grains; linolenic acid abundance increased simultaneously with linoleic acid decrease, showing disturbance in fatty acid synthesis and storage [85]. Later, the same group evaluated the impact of continuous generational exposure to 500 mg kg^−1^ nCeO_2_ on wheat grains and found that it affected DNA/RNA metabolites (e.g., thymidine, uracil, deoxyguanosine, adenosine monophosphate), as well as the levels of numerous metabolites such as nicotianamine, sugars (fructose-6-phosphate, glucose-6-phosphate, mannose-6-phosphate, hexose-6-phosphate, ribose, isomaltose and melibiose), sugar alcohols (erythritol, maltotriose, 6-deoxyglucitol, ribitol, 1-hexadecanol, and 6-deoxyglucitol), and diverse organic acids, such as fatty acids, among others [67,106]. In particular, the observed simultaneous decrease in nicotianamine, a metabolite involved in nutrient storage, with Fe decrease shows putative implications of nCeO_2_ exposure on grain nutritional value [67]. Interestingly, they also found that the impact of nCeO_2_ on metabolite levels/composition and plant growth depended on the offspring environment (e.g., N level in soil), modulating the influence of parental exposure [67,106]. These findings pinpoint that continuous exposure to nCeO_2_ and other NPs may have implications on ecosystem processes and that omics-based studies on generationally-exposed plants are key in deciphering the real impact on plants of NPs in soil and the environment.

#### 3.1.3. Silver Nanoparticles (nAg)

Despite not being the most produced worldwide, nanosilver has been considered the most used metal-based nanomaterial in consumer products [120]. Silver nanoparticles (nAg) are largely known and used due to their antimicrobial properties, leading to nAg use in medicine, cosmetics, pharmaceutics, agriculture, and textiles. However, nAg applications go beyond that, and they are also used in electronics, sensors, and solar cells [121]. In the agricultural sector, nAg have been explored as plant-stimulants [122], stress mitigators [123], and fungicides [124], despite the diverse studies also demonstrating their potential phytotoxicity [25,125,126]. The effects of nAg on plant metabolome are summarized in Table 3.

Under hydroponics, the foliar spray of *Rosmarinus officinalis* plants with AgNP at the concentrations 0, 25, 50, 100, and 200 ppm improved the levels of carnosic acid production, a metabolite related to antioxidant protection [72]. The treatment of *Trigonella foenum-graecum* L. plantlets with 1 mg L^−1^ of nAg prior to transplantation to soil played a dual role, increasing plant growth and the synthesis of diosgenin involved in stress response [70].

A recent study analyzed the impact of 10–40 mg L^−1^ bio-synthesized nAg on germination and early seedling development and metabolic profile in *T. aestivum*, incubating the seeds in the suspensions during the germination [71]. It was found that these NPs altered the primary metabolism, the mobilization of storage materials, sugars, and amino acid translocation from the endosperm to the seedlings. The nAg decreased the total content of soluble sugars, total amino acids, and total organic acids in coleoptile while increasing the content of total soluble sugars and decreased the total content of organic acids in roots; nAg decreased glucose and galactose, while increasing sucrose and 1-kestose in roots; in the coleoptile, glucose, fructose, and 1-kestose (40 mg L^−1^) were reduced whereas galactose increased, which may have led to the inhibition of coleoptile growth as galactose acts as a coleoptile growth inhibitor; in the endosperm, nAg decreased the levels of most carbohydrates, suggesting a reduction in starch mobilization in the endosperm induced by nAg, as well the reduction in the use of released sugars [71]. Apart from this, nAg also accumulated proline and aspartate/asparagine in roots, contrary to the coleoptile, which is in line with the rise of ROS in roots; in the TCA cycle, malate decreased in roots while citrate and fumarate increased; all identified organic acids decreased in the coleoptile, and only citrate and lactate were reduced in the endosperm [71]. In *T. aestivum* seedlings for which seeds had been treated with 10 mg L^−1^ nAg, changes in the three main groups of phytohormones, i.e., cytokinins, gibberellins, and auxins, were reported [81]. These authors described a high increment of GA_6_ and a strong decrease in cis-zeatin riboside in nAg treated plants. These changes were related to plant growth promotion and to the acceleration of the transition between the vegetative and reproductive stages, which coincided with the increase in sucrose, raffinose, and sorbitol, leading to superior yields [81]. 

In *C. sativus* plants grown in soil and treated with nAg (4 or 40 mg plant^−1^), it was demonstrated that foliar-sprayed NPs increased lipid peroxidation together with metabolite profile changes, with some of them being similar to those also induced by Ag^2+^ exposure while others were specific to the NPs, thus indicating a nanoscale size-specific effect [18]. Both Ag^2+^ and nAg increased metabolites related to antioxidant responses, for example, phytol, arbutin and salicin, 4-hydroxyquinazoline, and pyrogallol. Together with the up-regulation of sugars and sugar alcohols, these are important for stress-related responses [18]. Changes in fatty acids (e.g., pentadecanoic acid, linoleic and linolenic acids) also reveal membrane remodeling to adapt to adverse conditions, and the particular increase in salicylic acid highlights the broad defense-related response of cucumbers [18]. The nAg-specific-related response included the down-regulation of acetanilide, *p*-benzoquinone, 5,6-dihydrouracil, dibenzofuran, oxalic acid, oxamic acid, and lactamide, and the up-regulation of carbazole, raffinose, lactulose, citraconic acid, aspartic acid, dithioerythritol, D-erythronolactone, and *N*-methyl-L-glutamic acid [18]. In another study, the soil amendment with 12.5 mg kg^−1^ nAg up-regulated the tricarboxylic acid (TCA) cycle (e.g., malic acid and fumaric acid) and sugar metabolism (e.g., galactonic acid and tagatose) in *Arabidopsis thaliana*, increasing the energy and accelerating the stress response while reducing the plant growth [69]. On the other hand, the levels of amino acids (valine, serine, and aspartate) and the shikimate-phenylpropanoid biosynthesis were down-regulated, while some metabolites of the aspartate pathway increased, suggesting up-regulation of the aspartate family to feed the TCA cycle for energy supply [69]. Recently, it was found that foliar application of nAg (5, 7.5, and 10 mg L^−1^) can alter the essential oil composition of leaves and peels of lemon trees [127].

#### 3.1.4. Copper Nanoparticles (nCu)

Among the copper nanoparticles (nCu), copper oxide nanoparticles (nCuO) are the most common, being used in areas such as environmental remediation and environmental sensing, biomedical applications, agriculture, catalysis, electrochemistry, and energy storage, among others [128,129]. In agriculture, nCuO are commonly associated with their fungicidal and bactericidal properties and are highlighted as a more sustainable strategy for pest control when compared to their ionic or bulk counterparts [130,131,132]. Lately, the potential of nCuO/nCu for the mitigation of metal/metalloid toxicity in plants [133,134,135] and climate/environmental stresses such as drought [136,137] and salinity [138] has been explored. Nevertheless, nCuO show the potential to be phytotoxic [139] to several plant species, raising questions about their safety in agricultural systems. Among the species that showed some level of vulnerability to nCuO are *Vigna radiata* [140], *Glycine max* [141], *C. sativus* [142], *T. aestivum* [143], *Coriandrum sativum* [144], *Brassica rapa* [145], *Lactuca sativa* [146], and *O. sativa* [147]. Metabolic studies conducted with nCu in plants are still very limited, nevertheless those available suggest nCu’s ability to induce metabolic reconfiguration, as shown in Table 4.

In *C. sativus* grown in a hydroponic system, nCu at concentrations of 10 and 20 mg L^−1^ supplemented with a nutritive solution confirms that plant defense mechanisms against NPs-toxicity are related to several metabolic adjustments, in particular with an up-regulation of the metabolism of amino acids, amines, sugars, and carboxylic acids (4-aminobutyrate, acetylglucosamine, phenyllactate, nicotinurate, and glutaric acid monomethyl ester) in leaves [86]. Besides leaves, nCu exposure also changed the metabolite profile in root exudates, up-regulating several amino acids (alanine, ß-alanine, glycine, isoline, leucine, lysin, phenylalanine, proline, serine, threonine, and valine), salicylic and benzoic acids, pelargonic acid, while down-regulating citric acid and dehydroascorbic acid [86].

The active ingredient of the commercial nanopesticide Kocide 3000, nCu(OH)_2_, was tested in *T. aestivum* plants by foliar application of 100 mg L^−1^ nCu(OH)_2_ (as Cu content) [73]. The metabolomic analysis showed that nCu(OH)_2_ disturbed 34 metabolites in leaves and 27 in roots, with the great majority being down-regulated and a total of 17 pathways being perturbed in leaves and 17 in roots; glyoxylate/dicarboxylate metabolism and stilbenoid/diarylheptanoid/gingerol biosynthesis were disturbed only in leaves, while alterations in the glutathione metabolism and valine/leucine/isoleucine biosynthesis were specific to roots [73]. In another study, the effect of 10 and 100 mg nCu(OH)_2_ nanopesticide Kocide 3000 (leaf sprayed) on the levels of 12 low-molecular-weight antioxidants was evaluated in *Z. mays* plants grown in soil; the vitamins γ-tocopherol, α-tocopherol, and ascorbic acid, the nonprotein amino acid 4-aminobutyric acid, and the phenolic compound benzoic acid were not affected; nevertheless; the amino acids phenylalanine and tyrosine were up-regulated in response to 100 mg and proline was up-regulated in a dose-dependent manner, and the phenolic acids 1,3,4-benzenetriol, 4-hydroxycinnamic acid increased [75]. The metabolic changes observed indicate an up-regulation of phenolic biosynthesis as a protective mechanism against Cu-induced ROS, which may play a superior role in detoxifying ROS than enzymatic antioxidants [75]. Using the same nanopesticide, Zhao et al. [74] evaluated the leaf spray on *L. sativa* plants grown in hydroponics and found that together with K content increase, nCu(OH)_2_ nanopesticides decreased the total antioxidant capacity and altered 39 metabolites, including carboxylic acids (fumaric acid, aconitic acid, threonic acid, and oxalic acid were down-regulated, while malic acid and other 3 more were up-regulated), amino acids (alanine, GABA, oxyproline, and lysine were down-regulated and aspartic acid, glutamine, tryptophan, citrulline, glycine, and asparagine were up-regulated), and secondary metabolites such as polyamines and polyphenols (*cis*-caffeic acid, 3,4-dihydroxycinnamic acid, and chlorogenic acid decreased), and the vitamin dehydroascorbic acid (reduced). These findings showed that the application of nCu(OH)_2_ nanopesticides may affect the overall nutritional value of lettuce leaves, therefore the importance of defining the appropriate level of nCu(OH)_2_ to be used without decreasing crop nutritional value or limiting its antifungal activity [74].

#### 3.1.5. Zinc Oxide Nanoparticles (nZnO)

The antimicrobial properties of zinc oxide nanoparticles (nZnO) and their potential in the agricultural sector justify the high attention on these NPs; nevertheless, their potential as a nutrient fortifier and abiotic stress modulator also makes these NPs a subject of interest and research [148]. Indeed, diverse studies have reported nZnO capability in controlling several phytopathogens [149,150,151,152,153,154]. Zinc oxide nanoparticles (nZnO) have shown the ability to decrease environmental stresses on plants [22], such as metals [13,155,156], salt [84,157], and drought [158,159], and of being a nanofertilizer by slowly releasing Zn^2+^ [160,161]. Despite these benefits, nZnO may also be phytotoxic, mainly when high concentrations are used [76,148].

In terms of the impact of nZnO on the plant metabolome (Table 5), the foliar application of 50 mg L^−1^ nZnO on tobacco plants grown in hydroponics up-regulated30 metabolites (e.g., three alkaloids, nine flavonoids, and three phenylpropanoids) and down-regulated 22 metabolites (e.g., one alkaloid, three flavonoids, five amino acids and derivatives) in roots, while in leaves 12 metabolites were increased (e.g., alkaloids, four amino acids and their derivatives, one flavonoid) and 17 were reduced (e.g., two alkaloids, five flavonoids, two nucleotides, and their derivates) [13].

On the other hand, the same species treated with 400 mg kg^−1^ soil of nZnO increased globulin content, while 800 mg kg^−1^ enhanced glutelin content, both proteins responsive to (a)biotic stress [116]. Li et al. [65] reported that the foliar application of nZnO in *C. sativus* at concentrations of 100 mg L^−1^ increased the contents of several metabolites, leading to growth-promoting effects and at the same time increasing defense and stress responses. For instance, nZnO changed the metabolism of the amino acids in leaves, increasing the contents of methionine, tryptamine, tryptophan, isoleucine, valine, phenylalanine, and tyrosine, while in roots it negatively correlated with tryptophan and up-regulated tyrosine. Beside the biosynthesis of the amino acids, these NPs also modulated the carbon metabolism (increased the levels of e.g., 4-hydroxyproline galactoside and L-galacto-2-heptulose, while reducing the levels of e.g., dihydrozeatin-*O*-glucoside in leaves; in roots most of the compounds were down-regulated) and down-regulated the flavonoid pathway in leaves (decreased the levels of isorhamnetin 3-rutinoside 4′-rhamnoside, apigenin 7-rhamnoside-4′-rutinoside, quercetin, rutin, and quercetin 3-*O*-glucoside) [65]. The production of some organic acids, such as salicylic acid, was also stimulated in leaves, while in roots all were down-regulated by these NPs in *C. sativus*. In hydroponics, foliar spray of *Sophora alopecuroides* seedlings with 50 or 100 mg L^−1^ nZnO increased the levels of dehydroascorbic acid and malonic acid and decreased the amino acids asparagine, tryptophan, phenylalanine, and *N*-methyl-DL-alanine in leaves. In roots, nine metabolites were increased (e.g., L-malic acid, gentiobiose, 3,6-anhydro-D-galactose, ribose, α-ketoglutaric acid, 4-hydroxybutyrate, and ascorbate) and nine also decreased, including the amino acids asparagine, aspartic acid, acycloleucine, L-allothreonine, 3-cyanoalanine, and tryptophan, lactose and meliobiose [84].

In *Phaseolus vulgaris* grown in soil, plants were foliar sprayed or irrigated with nZnO suspensions (250, 500, 1000, and 2000 mg L^−1^) and revealed that the mode of application and the dose differently affected the morphology and physiology of the plants, e.g., photosynthesis, the enzymatic antioxidant response, and the proline level (increased in a dose-dependent manner when plants were foliar sprayed, while decreased or was not altered in soil application) [76]. The metabolomic analysis was performed only in plants treated with the highest dose. It revealed that the application method differently affected the metabolite profile: Foliar spray down-regulated seven metabolites, including flavonoids such as 2′-*O*-methylisoliquiritigenin, pinostrobin, and (−)-medicarpin, and the terpene oleanolate 3-β-D-glucuronoside-28-glucoside, while it up-regulated 13 metabolites such as lipids (e.g., (4*Z*,7*Z*,10*Z*,13*Z*,16*Z*,19*Z*)-docosahexaenoate and sphinganine 1-phosphate), phenolics such as salicin and humulone, and the hormones gibberellins and indole-3-butyryl glucose. Root applications did not negatively affect the metabolite contents and even up-regulated diverse carotenoids, flavonoids such as luteolin, kaempferol, bracteatin, tricetin, quercetin, delphinidin and 4′-methoxyisoflavan-2′,4,7-triol, the hormone jasmonic acid, and shikonin [76]. These results showed that foliar application targeted photosynthesis and had a dose-dependent effect, contrary to root irrigation, which did not show dose-related responses and targeted the antioxidant response [76]. It is noteworthy that this study also highlighted that nZnO and ZnSO_4_ induced distinct toxic effects on plants [76].

#### 3.1.6. Magnetic Iron Oxide Nanoparticles (nFe_3_O_4_)

Magnetic iron oxide nanoparticles (nFe_3_O_4_) are widely used in catalysis, medical science (e.g., magnetic resonance imaging, biosensor, drug delivery, etc.), separation technology, in the agri-food sector (e.g., fertilizer, antimicrobial, slow-release system, additives, protein immobilization, storage, etc.), and environmental/water remediation [5,162,163]. In terms of the interaction of nFe_3_O_4_ with plants, improvement in plant growth [164,165,166] and photosynthesis [167,168], antioxidant response activation, defense response elicitation against viruses [169] and fungi [170], metal toxicity mitigation [13,167,171,172], drought [173], salinity [174], and Fe-deficiency stress alleviation [175] have been reported. Nevertheless, it should not be forgotten that as with other NPs, under some conditions, nFe_3_O_4_ can also be detrimental to plant species [175,176].

Much of the physiological alterations induced by these NPs result from induced metabolic rearrangements, as can be confirmed by the still scarce metabolomic studies available with nFe_3_O_4_ in plants (Table 6). For example, the application of nFe_3_O_4_ on *Nicotiana tabacum* plants grown hydroponically up-regulated 16 metabolites (e.g., perakine– alkaloid, farrerol and quercetin–flavonoids, and *p*-coumaryl alcohol–phenylpropanoids) and down-regulated 24 metabolites (e.g., riddeline, echimidine, 3-(carboxymethylamino)propanoic acid–alkaloids, cyanin, (+)-afzelechin, hyperoside–flavonoids, 3-hydroxy-4-methoxycinnamic acid–phenylpropanoid) in roots, while in leaves 26 metabolites were increased (e.g., 5 alkaloids, cysteinylglycine, glutathione, L-homoglutamic acid, L-homoserine, L-threonine, L-arginine, 4-aminobutyric acid–amino acids and their derivatives, three flavonoids) and 19 reduced (e.g., two alkaloids, two flavonoids, one phenylpropanoid) [13].

Zhao et al. [19] reported the metabolome readjustments induced by nFe_3_O_4_ at the concentration of 100 mg kg^−1^ soil, in both leaves and roots of *Z. mays*, despite being more pronounced in leaves. In leaves, 11 pathways were altered, including those related to nitrogen metabolism (particularly the amino acids serine, tyrosine, valine, threonine, isoleucine, phenylalanine, glutamic acid, proline, and glutamine), TCA cycle, glycolysis and gluconeogenesis, and pyrimidine metabolism, in addition to the phenylalanine and tyrosine up-regulation [19]. On the other hand, significantly fewer pathways were disrupted in roots, accounting for only four: inositol phosphate metabolism, glycerolipid metabolism, ascorbate and aldarate metabolism, and the TCA cycle. The up-regulation of several ROS scavengers (1-hydroxyanthraquinone, 4-hydroxycinnamic acid, caffeic acid, and ascorbate) was detected, as well as phenylalanine and tyrosine, also involved in defense responses as precursors of defense-related secondary metabolites, which altogether suggest that nFe_3_O_4_ induced a significant stress response [19]. In *Z. mays* plants grown in soil, smaller doses of nFe_3_O_4_ (50 mg kg^−1^ soil) were able to re-program the root metabolome despite no phenotypic changes being observed [83]. Among the 191 metabolites identified, 20 were significantly altered and all of them were down-regulated, including sugars (e.g., xylose, galactinol, levoglucosan, 1,5-anhydroglucitol, myo-inositol, and threitol), amino acids (e.g., glutamine, glutamic acid and proline), phenolics related with the antioxidant response (catechin, gallocatechin, benzoic acid, and hydroxybenzoic acid), and organic acids (e.g., fumaric acid). These changes reflected alterations in several metabolic pathways: four of them were related to sugar metabolism, with particular relevance of the inositol phosphate metabolism; five of them were related to amino acid metabolism, indicating the re-programming of nitrogen metabolism to manage plant growth instead of a stress response; one of them was related to oxidative stress, the ascorbate and aldarate metabolic pathway [83]. Overall, in this case, it seems that nFe_3_O_4_ had a protective effect on *Z. mays*.

### 3.2. Metabolomics to Understand the Stress Tolerance Promotion

Concerning stress tolerance improvement, several studies highlighted the important role of NPs in promoting plant defense under heavy metal, salinity, and drought conditions. For instance, the exposure of *Z. mays* plants (grown in hydroponics) to nTiO_2_ (100 or 250 mg L^−1^) via leaves exerted higher influence on the metabolic profile of maize than the application via roots; it also had a stronger impact in alleviating Cd-induced (50 µM) toxicity [104] (Table 1). The Cd tolerance was related with the activation of antioxidant responses, synthesis of antioxidants (alanine, aspartate, and glutamate metabolism, as well as glycine, serine, and threonine metabolism), and regulation of major metabolic pathways towards the energy metabolism such as the galactose metabolism and citrate cycle [104]. In rice, the content of hormones such as indole-3-acetic acid (IAA), methyl jasmonate (JA), isopentenyl adenosine, and zeatin riboside were altered when treated with nTiO_2_ (10–1000 mg L^−1^) in the presence of Cd (10 or 20 mg L^−1^) [93] (Table 1). The foliar application of 50 mg L^−1^ Fe_3_O_4_ and nZnO (Table 5 and Table 6) showed great potential in alleviating Cd toxicity in tobacco seedlings [13] under Cd-induced stress. These NPs stimulated the accumulation of several metabolites, such as flavonoids (quercetin, isorhamnetin, isoquercitrin), alkaloids (denudatine), terpenes (catalpalactone, kaurenoic acid, and limonin), and amino acids (*N*6-acetyl-L-lysine and L-theanine) that were positively correlated with improved plant growth (plant height and shoot fresh weight) under Cd-stress conditions.

As Wan et al. [84] demonstrated, nZnO also contributes to salt tolerance (Table 5). In *Sophora alopecuroides* seedlings, the foliar application of 50 or 100 mg L^−1^ nZnO with a size of 20–45 nm improves growth by modulating metabolite profile (increase in lactobionic acid, 4-hydroxypyridine, phenyl beta-D-glucopyranoside, glucose-6-phosphate, and L-malic acid in leaves and roots). Moreover, the increase in glucose-6-phosphate, L-malic acid, fumaric acid, and succinic acid in the leaves, and aconitic acid, citric acid, fructose-6-phosphate, pyruvic acid, and α-ketoglutaric acid in the roots suggested that foliar application of ZnO NPs promoted glycolysis and the TCA cycle to generate more energy [84]. The lipid pathway is also modulated by ZnO NPs in response to salinity, inducing the accumulation of linolenic acid in the leaves which contributes to maintaining membrane integrity under salt stress. The application of nTiO_2_ in roots (100 and 200 mg L^−1^) modulates the growth and secondary metabolism of *Dracocephalum moldavica* L. in response to salt stress [105] (Table 1). These NPs decreased the negative effects of salt stress, increasing agronomic traits and the amount of geraniol, a stress signaling molecule that regulates the expression of several genes related to plant stress defense [105,177].

Plant drought negative effects can also be ameliorated by nTiO_2_ (Table 1), nZnO (Table 5), and nMg. The application of nTiO_2_ (10 and 100 mg L^−1^) on leaves of *Stevia rebaudiana* under water limitations induced a stress response by increasing proline levels, apart from up-regulating rebaudioside A and stevioside steviol glucosides [103]. In maize plants under drought conditions, the root application of nZnO (100 mg L^−1^) promoted the melatonin synthesis and activated the antioxidant enzyme system, alleviating the drought-induced damage to mitochondria and chloroplast [82]. Melatonin levels are essential in regulating plant growth and morphogenesis and in the defense against abiotic stress. In *Z. mays*, nZnO (100 mg L^−1^) also alleviated drought stress by promoting starch and sucrose biosynthesis and glycolysis metabolism and endorsed drought tolerance by increasing water use efficiency and photosynthesis [158]. In *Achillea millefolium*, nMg foliar application at the concentration of 0.1, 0.3, and 0.5 g L^−1^ increased the levels of several secondary metabolites (sesquiterpenes), a strategy to protect from the harmful effects of drought stress [14].

## 4. Conclusions

All these studies demonstrated that metabolomic analysis is a powerful and needed tool to understand the mode of action of metal-NPs on plants and the molecular mechanisms involved in plant response to putative NP-induced stress. This mechanistic understanding of NP action is crucial for evaluating the risk of these materials in the environment and to develop sustainable and safe nano-strategies for plant protection and fortification to be further applied in agriculture. It became clear that metal-NPs induce ROS accumulation and re-program several plant metabolic pathways, readjusting the levels of several metabolites that promote stress tolerance and growth. The action mechanism of NPs involves stimulating the plant antioxidant defense system (increasing the synthesis of antioxidants and antioxidant enzymes and the upregulation of stress-related genes), helping plants overcome the adverse effects of NP-induced stress. Moreover, NPs regulate carbohydrate (e.g., sucrose and starch biosynthesis) and nitrogen metabolism, modulate hormone profile (e.g., IAA and methyl JA), and alter the TCA cycle and lipid pathway. However, applying NPs as an elicitor requires in-depth knowledge to identify all the potential effects on humans and ecosystems. Moreover, field studies considering the interaction of several stress factors and NPs are necessary to define doses, time, and type of exposure, among other things.

## Figures and Tables

**Table 1:** Main Effects of nTiO_2_ on plant metabolome.

Particle Primary Size (nm)	Concentration	Species	Application Method	Growth Medium	Main Metabolic Effects	Ref.
21	5, 50 and 150 mg L^−1^	Wheat	Together with nutritive solution	Hydroponics	Up-regulated the aspartate pathway, serine, alanine and valine metabolisms, and the glycerolipids’ biosynthesis; down-regulated citrate and glyoxylate metabolisms	[78]
20	100, 250 and 500 mg L^−1^	Rice	Together with nutritive solution	Hydroponics	Increased glucose-6-phosphate, glucose-1-phosphate, succinic and isocitric acid concentration; up-regulated the tricarboxylic acid cycle and the pentose phosphate pathway; increased fatty acids, amino acids and secondary metabolites; decreased sucrose, isomaltulose, and glyoxylic acid levels; down-regulated arbohydrate synthesis metabolism including starch and sucrose metabolism, and glyoxylate and dicarboxylate metabolism	[77]
20	25, 50, 150, 250, 500, and 750 mg kg^−1^	Rice	Soil amendment	Soil	750 mg kg^−1^ increased the levels of proline, aspartic acid, glutamic acid, palmitic acid, glycerol, inositol, ribitol, phosphoric acid, glycerol-3-phosphate proline, aspartic acid and glutamic acid; decreased the levels of overall fatty acids (linoleic and oleic acids), organic acids and sugars	[68]
5–10	100 mg kg^−1^	Maize	Soil amendment	Soil	Increased 4-aminobutyric acid, succinate semialdeyde, putrescine, α-ketoglutaric acid, hexenedioic acid, threonic acid, 2-ketobutiric acid, palatinito and, 1-hydroxyanthraquinone; decreased succinic acid, benzoylformic acid, manose, phosphate and adenine	[19]
5–10	2.7 and 27 mg plant^−1^	Cucumber	Foliar spray	Soil	Changed the profile of 31 metabolites: increased the abundance of amino acids such as 3-hydroxy-L-proline, norvaline, *N*-ethylglycine, glutamine, 5-aminovaleric acid, and phenylacetaldehyde; increased 2-ketobutyric acid, 3-hydroxyflavone, 2-furoic acid, phytanic acid, and pelargonic acid; decreased *N*-acetyl-5-hydroxytryptamine and 2-amino-2-norbornanecarboxylic acid levels; reduced succinic acid, tartaric acid and glucoheptonic acid	[80]
25	10 and 100 mg L^−1^	Stevia	Together with nutritive solution	Hydroponics	Increased proline levels; up-regulated rebaudioside A and stevioside glucosides	[103]
6.5	100 and 250 mg L^−1^ along with 50 µM Cd	Maize	Together with nutritive solution or foliar sprayed	Hydroponics	Up-regulated alanine, aspartate and glutamate metabolism, as well as glycine, serine and threonine metabolism, galactose metabolism and citrate cycle; promoted Cd tolerance	[104]
21	10–1000 mg L^−1^ along with Cd (10 and 20 mg L^−1^)	Rice	Nutritive solution	Hydroponics	Altered the profile of indole-3-acetic acid, methyl jasmonate, isopentenyl adenosine, and zeatin riboside	[93]
20–30	100 and 200 mg L^−1^	Moldavian balm	Root application	Soil	Decreased geraniol levels	[105]

**Table 2:** Main Effects of nCeO_2_ on plant metabolome.

Particle Size (nm)	Concentration	Species	Application Method	Growth Medium	Main Metabolic Effects	Ref.
231	62.5, 125, 250, and 500 mg L^−1^	Rice	Seed application	Petri dishes	Increased lauric (at 62.5 and 125 mg L^−1^) and valeric (150 mg L^−1^) acids, and 500 mg L^−1^ reduced lignin; decreased the content fatty acids (palmitic, oleic, stearic, linoleic and linolenic), and myristic acid	[66]
231	125, 250, and 500 mg kg^−1^	Wheat	Soil amendment	Soil	125 mg kg^−1^ increased the content of amino acids (arginine, aspartic acid, glycine, histidine, and lysine) and linolenic acid; decreased linoleic acid decrease	[85]
231	500 mg kg^−1^	Wheat	Soil amendment	Soil	Generationally-exposed plants showed alteration in 180 metabolite levels, including DNA/RNA metabolites (thymidine, uracil, adenosine monophosphate and deoxyguanosine), sugars and sugar alcohols, organic acids, among others; decreased nicotianamine and Fe simultaneously; the changes were dependent on N content in soil	[67,106]
10–30	25, 50, and 100 mg L^−1^	Bean	Foliar spray	Agar	Increased of terpenes and terpenoids (e.g., steroids, steroid hormones, di and triterpenes, and carotenoids); alkaloids such as 1-*O*-caffeoyl-*ß*-D-glucose, esculin, and (+)-sesamolinol; flavonoids profile was altered and was dose-dependent; accumulation of compounds involved in the glucosinolate pathway and phytoalexin biosynthesis; increased gibberellins, auxins and brassinosteroids	[79]
10	400 and 800 mg Kg^−1^	Cucumber	Soil	Soil amended	400 mg kg^−1^ increased globulin and decreased glutelin; 800 mg kg^−1^ decreased phenolic content and increase of total sugar content	[116]

**Table 3:** Main Effects of nAg on plant metabolome.

Particle Size (nm)	Concentration	Species	Application Method	Growth Medium	Main Metabolic Effects	Ref.
<100	25, 50, 100, and 200 ppm	Rosemary	Foliar spray	Hydroponics	Increased carnosic acid production	[72]
8–21	1 μg mL^−1^	Fenugreek	Seed application	Soil	Increased the production of diosgenin	[70]
5–10	10, 20 and 40 mg L^−1^	Wheat	Seed application	Petri dishes	Decreased glucose, galactose and malate, and increased sucrose, 1-kestose, citrate, fumarate, proline and aspartate/asparagine in roots; decreased glucose, fructose, and 1-kestose (40 mg L^−1^) in coleoptile, but increased galactose; decreased carbohydrates in the endosperm and citrate and lactate	[71]
29	4 and 40 mg plant ^−1^	Cucumber	Foliar spray	Soil	Down-regulated acetanilide, *p*-benzoquinone, 5,6-dihydrouracil, dibenzofuran, oxalic acid, oxamic acid, and lactamide, and up-regulated carbazole, raffinose, lactulose, citraconic acid, aspartic acid, dithioerythritol, D-erythronolactone, and *N*-methyl-L-glutamic acid contents; decreased linoleic and linolenic acids, and increased pentadecanoic acid	[18]
13–15	10 mg L^−1^	Wheat	Seed incubation during germination	Hydroponics + soil	Altered the phytohormones profile and proportion: increased GA_6_ and decreased cis-zeatin riboside; increased the content of transport of sugars such as sucrose, raffinose, and sorbitol	[81]
10	12.5 mg kg^−1^	Arabidopsis	Soil amendment	Soil	Increased TCA (e.g., malic, fumaric and threonic acids) and sugar metabolism (e.g., galactonic acid and tagatose), decreased amino acids (valine, serine, and aspartate), sugar alcohols (e.g., glycerol and ribitol), and shikimate-phenylpropanoid-related metabolites (e.g., scopoletin and melatonin)	[69]

**Table 4:** Main Effects of nCu on plant metabolome.

Particle Size (nm)	Concentration	Species	Application Method	Growth Medium	Main Metabolic Effects	Ref.
40	10 and 20 mg L^−1^	Cucumber	Together with nutritive solution	Hydroponics	In leaves, up-regulated 4-aminobutyrate, acetylglucosamine, phenyllactate, nicotinurate and glutaric acid monomethyl ester, and down-regulated *N*-carbamoylaspartate desaminotyrosine, *N*-acetyltyrosine, *N*-carbamoyl-beta-alanine, thymidine, cytidine, melatonin, kynurenine, caprate, *O*-acetylcarnitine, carnosine, NADH, ribofavin, urea, epicatechin, chlorogenate; altered the metabolite profile of root exudates: increased amino acids (alanine, ß-alanine, glycine, isoline, leucine, lysin, phenylalanine, proline, serine, threonine and valine), salycilic and benzoic acids, pelargonic acid, and decreased citric acid and dehydroascorbic acid	[86]
50	100 mL L^−1^	Wheat	Foliar application	Water- saturated vermiculite	Disturbed 34 metabolites in leaves and 27 in roots, mostly down-regulated, with a total of 17 pathways being perturbed in leaves and 17 in roots; glyoxylate/dicarboxylate metabolism and stilbenoid/diarylheptanoid/gingerol biosynthesis were disturbed in leaves, and glutathione metabolism and valine/leucine/isoleucine biosynthesis in roots	[73]
~50 to > 1000	10 and 100 mg	Maize	Foliar spray	Soil	Increased phenolic acids 1,3,4-benzenetriol, 4-hydroxycinnamic acid; 100 mg increased amino acids phenylalanine, tyrosine and proline	[75]
~50 to > 1000	1050 and 1555 mg L^−1^	Lettuce	Foliar spray	Hydroponics	Decreased total antioxidant capacity; altered 39 metabolites, carboxylic acids (decreased fumaric acid, aconitic acid, threonic acid and oxalic acid, and increased malic acid), amino acids (decreased alanine, GABA, oxoparoline and lysine, and increased aspartic acid, glutamine, tryptophan, citrulline, glycine, and asparagine), and secondary metabolites such as polyamines, polyphenols (*cis*-caffeic acid, 3,4-dihydroxy-cinnamic acid and chlorogenic acid decreased) and the vitamin dehydroascorbic acid (reduced)	[74]

**Table 5:** Main Effects of nZnO on plant metabolome.

Particle Size (nm)	Concentration	Species	Application Method	Growth Medium	Main Metabolic Effects	Ref.
~30	50 mg L^−1^	Tobacco	Foliar spray	Hydroponics	Increased 30 metabolites (e.g., three alkaloids, nine flavonoids, and three phenylpropanoids) and decreased 22 metabolites (e.g., one alkaloid, three flavonoids, five amino acids and derivatives) in roots; in leaves increased 12 metabolites (e.g., alkaloids, four amino acids and their derivatives, one flavonoid) and decreased 17 metabolites (e.g., two alkaloids, five flavonoids, two nucleotides and their derivates)	[13]
10	400 and 800 mg kg^−1^	Cucumber	Soil	Soil	400 mg kg^−1^ increased globulin content, while 800 mg kg^−1^ increased glutelin	[116]
20	100 mg mL^−1^	Cucumber	Foliar spray	Soil	Increased methionine, tryptamine, tryptophan, isoleucine, valine, phenylalanine, and tyrosine in leaves, while in roots decreased tryptophan and up-regulated tyrosine; in leaves increased 4-hydroxyproline galactoside and L-galacto-2-heptulose, and decreased dihydrozeatin-*O*-glucoside, isorhamnetin 3-rutinoside 4′-rhamnoside, apigenin 7-rhamnoside-4′-rutinoside, quercetin, rutin, and quercetin 3-*O*-glucoside	[65]
20–45	50 or 100 mg L^−1^	Kudouzi	Foliar spray	Hydroponics	Increased dehydroascorbic acid and malonic acid and decreased the amino acids asparagine, tryptophan, phenylalanine and *N*-methyl-DL-alanine in leaves; in roots increased L-malic acid, gentiobiose, 3,6-anhydro-D-galactose, ribose, α-ketoglutaric acid, 4-hydroxybutyrate, and ascorbate, and decreased asparagine, aspartic acid, acycloleucine, L-allothreonine, 3-cyanoalanine, tryptophan, lactose and meliobiose	[84]
10–30	250, 500, 1000 and 2000 mg L^−1^	Bean	Foliar spray or irrigated	Soil	Foliar application (2000 mg L^−1^) decreased flavonoids (e.g., 2′-*O*-methylisoliquiritigenin, pinostrobin, and (—)-medicarpin, and the terpene oleanolate 3-β-D-glucuronoside-28-glucoside) and increased lipids (e.g., (4*Z*,7*Z*,10*Z*,13*Z*,16*Z*,19*Z*)-docosahexaenoate and sphinganine 1-phosphate), phenolics such as salicin and humulone, and the hormones gibberellins and indole-3-butyryl glucose; root application increased carotenoids, flavonoids (e.g., luteolin, kaempferol, bracteatin, tricetin, quercetin, delphinidin and 4′-methoxyisoflavan-2′,4,7-triol), jasmonic acid and shikonin	[76]
20	100 mg L^−1^	Maize	Root application	Soil	Increased the production of melatonin, starch and sugars biosynthesis, and glycolysis metabolism	[82]

**Table 6:** Main Effects of Fe_3_O_4_ on plant metabolome.

Particle Size (nm)	Concentration	Species	Application Method	Growth Medium	Main Metabolic Effects	Ref.
20	50 mg L^−1^	Tobacco	Foliar spray	Hydroponics	Increased 16 metabolites (such as perakine–alkaloid, farrerol and quercetin–flavonoids, and ρ-coumaryl alcohol–phenylpropanoids) and decreased 24 metabolites (such as riddeline, echimidine, 3-(carboxymethylamino)propanoic acid–alkaloids, cyanin, (+)-afzelechin, hyperoside–flavonoids, 3-hydroxy-4-methoxycinnamic acid–phenylpropanoid) in roots; in leaves increased 26 metabolites (such as five alkaloids, cysteinylglycine, glutathione, L-homoglutamic acid, L-homoserine, L-threonine, L-arginine, 4-aminobutyric acid–amino acids and their derivatives, three flavonoids) and decreased 19 metabolites (such as two alkaloids, two flavonoids, one phenylpropanoid)	[13]
30	100 mg Kg^−1^	Maize	Soil amendment	Soil	In leaves, 11 pathways were altered, nitrogen (particularly the amino acids serine, tyrosine, valine, threonine, isoleucine, phenylalanine, glutamic acid, proline and glutamine), TCA cycle, glycolysis and gluconeogenesis, and pyrimidine metabolism; in roots the pathways related to inositol phosphate metabolism, glycerolipid metabolism, ascorbate and aldarate metabolism, and TCA cycle were disrupted, increased 1-hydroxyanthraquinone, 4-hydroxycinnamic acid, caffeic acid, ascorbate, phenylalanine and tyrosine	[19]
30	50 mg Kg^−1^	Maize	Irrigation water	Soil	Decreased 20 metabolites, including sugars (e.g., xylose, galactinol, levoglucosan, 1,5-anhydroglucitol, myo-inositol, and threitol), amino acids (e.g., glutamine, glutamic acid and proline), phenolics (catechin, gallocatechin, benzoic acid, hydroxybenzoic acid), organic acids (e.g., fumaric acid), and aldarate metabolic pathway	[83]

## Data Availability

Not applicable.
